# Short-Term and Long-Term Mechanical Properties of Lightweight Concrete with Sintered Aggregate

**DOI:** 10.3390/ma18132977

**Published:** 2025-06-23

**Authors:** Paweł M. Lewiński, Zbigniew Fedorczyk, Przemysław Więch, Łukasz Zacharski

**Affiliations:** Building Research Institute (ITB), Filtrowa 1, 00-611 Warsaw, Poland; z.fedorczyk@itb.pl (Z.F.); p.wiech@itb.pl (P.W.); l.zacharski@itb.pl (Ł.Z.)

**Keywords:** lightweight aggregate concrete, lightweight sintered aggregate, short-term tests, long-term tests

## Abstract

The aim of this work is to determine the short-term and long-term mechanical properties of lightweight concrete with relatively new sintered aggregate, as knowledge of these parameters is essential to the design of prestressed structures. The problem can be placed in a broader ecological context, because the aggregate comes from recycled power plant ash. This research study was planned based on two concrete mixtures that were already used in previous publications, as the aim of this work was to conduct comparative research by using other methods. In particular, the aim was to investigate the long-term properties of lightweight concrete by using standard methods and appropriate equipment, such as creep-testing machines. As a result of these studies, the secant modulus of elasticity, cylindrical strength, cubic strength, axial tensile strength, splitting tensile strength, bending strength, and shrinkage and creep strain were determined. This study confirmed the short-term properties of concrete obtained in previous studies but did not confirm the results regarding shrinkage and creep. These results turned out to be much higher, which means that these values should not be tested by non-standard methods. An unusual process of development of the elastic modulus and axial tensile strength was observed, and the reasons for these phenomena were described.

## 1. Introduction

The issue of using concrete with lightweight sintered aggregate (LSA) for prestressed concrete structures has been the subject of works by many authors in recent years. Lightweight concretes constitute a group of materials with different properties. Thanks to the use of lightweight concrete with sintered aggregate as a material that guarantees the reduction in the dead weight of the structure while maintaining the required strength properties of concrete, it turns out that in prestressed structures, additional capacity and deflection reserve are obtained. Due to its properties, lightweight aggregate concrete (LWAC) has been widely applied in the construction industry, including high-rise buildings, prefabricated structures, bridges, and oil platforms.

However, lightweight aggregate concrete is still a problematic material for use in construction. Currently, there are not many standards for testing lightweight concrete. There is little knowledge about such materials, and the lack of research discourages designers from using them in projects. Therefore, the reliable testing of the long-term properties of the concrete under consideration is a prerequisite for these new applications.

The characteristics of the most common lightweight aggregates on the Polish construction market are listed in Table 1 and Table 2.

Until now, artificial aggregates such as Pollytag and Certyd made of sintered fly ash were available, but the production of Pollytag aggregate was stopped in 2015. Keramzyt aggregate is also no longer produced in Mszczonów. In turn, Certyd aggregate has been produced since 2015 and has been implemented in design and construction practice as a result of a series of research studies, which are discussed below. Although the properties of Certyd and Pollytag are comparable, they are produced using different technologies, but the production of Pollytag aggregate is no longer profitable. Certyd aggregate is produced in Sowlany, near Białystok, using LSA technology. The lightweight, high-strength sintered aggregate used for the concrete under study is a ceramic, ash-porite aggregate produced using domestic technology. It is lightweight and porous and has high mechanical strength, as its resistance (strength) to crushing, similarly to Pollytag aggregates, can exceed 6.0 Mpa (depending on its fraction), although the manufacturer currently guarantees crushing resistance for a fraction of 4/10 above 4 Mpa. Other general properties of the Certyd aggregate with a fraction of 4/10 (adopted in this work), apart from the properties given in Table 1 and Table 2, include an average bulk density of about 700 kg/m^3^, a grain density of about 1300 kg/m^3^, a water absorption after 24 h of about 20%, frost resistance ≥ 5%, chloride content ≤ 0.05%, acid-soluble sulfate content ≤ 1.0%, total sulfur content ≤ 1.0%, and thermal conductivity coefficient *λ* = 0.14 W/m∙K (dry aggregate).

The main raw material for aggregate production Is the ash from the combustion of hard coal in fine coal boilers [1] in power plants. As a result, the final product comes from recycling ashes, which provides a granulate from which the aggregate is produced after processing. As a recycled product, it can be considered environmentally friendly. In addition, the sintering process makes the aggregate less absorbent and frost-resistant. Lightweight aggregate with high strength is manufactured according to domestic technology. It is created as a result of the high-temperature sintering (1000–1200 °C) of properly prepared anthropogenic minerals in rotary kilns under controlled conditions, followed by fractionation and possibly crushing. Since lightweight concretes are a group of materials with various properties, their mechanical behavior requires careful verification. Lightweight concretes are characterized by greater structural homogeneity than plain concretes, resulting from the tight construction of the contact zone between the grout and aggregate. This ensures, among others, the regular grain shape of artificial aggregates.

Due to the different structures, lightweight concretes usually behave differently under load and show a different failure mechanism compared with plain concretes. Tests [2] showed that for concretes with aggregate made of ashes, the straight-line course of the *σ*–*ε* relationship reaches up to 90% of the ultimate stress. In concretes with lightweight aggregate, the high elastic energy stored in them during loading causes the rapid propagation of cracks, which irreversibly leads to the sudden destruction of the material. However, the first load cracks appear only when the load-bearing capacity is exhausted by about 85–90%. In the case of plain concretes, destruction usually occurs in the contact zone of the aggregate with the grout. In lightweight concretes, the failure crack usually runs through the aggregate, because in these concretes, the aggregate is the weakest element of the composite structure. Therefore, lightweight concretes are more brittle. With the same proportions of the components of the mix of plain and lightweight concrete, in the case of lightweight concrete, a higher cement class is required to obtain the same strength class. In the case of lightweight concrete, the strength is influenced by the same properties as in the case of plain concrete, i.e., the W/C ratio, the cement content, and the age of the concrete. Nevertheless, due to the high absorption of mixing water by the aggregate, it is difficult to estimate the total effect of the W/C ratio on the value of concrete compressive strength. The Young’s modulus of concrete, which is a two-component composite, depends on the parameters of both the aggregate and the grout, their volumetric shares, and mutual adhesion. Due to the lower density compared with plain concrete, lightweight concrete is also characterized by a lower secant modulus of elasticity.

Following the launch of Certyd aggregate production in 2015, significant publications and monographs on the properties of concrete with lightweight sintered aggregate of the considered type, resulting from research conducted, have been published since 2016, among others, by the ImiKB laboratory of Cracow University of Technology. The purpose of these tests has been to determine the mechanical properties of concrete, i.e., the compressive strength, tensile strength, and modulus of elasticity, as well as determining the development of shrinkage and creep in concrete. The results of these tests show that relatively high compressive strength of concrete can be obtained without much difficulty, so it can be used in prestressed structures. The monograph [2] presents the state of the art for structural lightweight concretes. The main part of the monograph is a description of structural lightweight concrete properties with respect to their complex modeling. The work also discusses the possibility of the modification of lightweight concrete properties with admixtures, additives, polymers, and fibers. The works by Domagała et al. [2,3,4,5,6] included the results of many studies on the influence of various factors on the mechanical properties of lightweight concrete, such as aggregate impregnation and scale effect. Many works have been devoted to the use of LWAC for the construction of prestressed slab floors [7,8,9,10,11,12,13,14,15,16]. The papers [8,9,13] discuss in detail tests of LWAC with Certyd aggregate in terms of determining shrinkage and creep strain. The results of these tests indicate a low creep coefficient of lightweight concrete with sintered aggregate. The works by Szydłowski et al. [9,11,12,13,14,15,16] represent an important contribution to the basics of designing prestressed concrete structures made with the use of lightweight sintered aggregate. The article by Małaszkiewicz and Jastrzębski [17] presents research results assessing the possibility of making lightweight self-compacting concrete from local sintered fly ash aggregate. Previously, Kaszyńska and Zieliński [18] studied the effect of lightweight aggregate on minimizing the autogenous shrinkage of self-consolidating concrete.

The Influence of various properties of lightweight aggregates (physical, chemical, etc.) on the mechanical properties of concrete made using these aggregates has been described based on extensive experimental studies, among others, in the works [19,20,21,22,23]. Shrinkage phenomena of LWAC, including shrinkage cracks, were analyzed in a number of papers, including [24,25,26], and the phenomenon of early thermal-shrinkage stresses resulting from the release of hydration heat during the setting process of LWACs was analyzed in the works [27,28,29]. The simultaneous creep and shrinkage action on LWAC has also been the subject of several works, including [21,22,30,31,32,33,34,35]. A number of physicochemical analyses of LWAC with aggregate produced according to the LSA technology (Białystok, Poland), along with comparisons in the scope of applications of other lightweight artificial aggregates, were conducted by researchers from Białystok University of Technology (cf. works [36,37,38,39,40,41]), Wrocław University of Science and Technology, and Warsaw University of Technology in Płock (cf. [42]) (and also from LSA company (cf. [43,44,45]). Extensive studies on floor elements made using LWACs with the discussed aggregate were conducted by scientists from Łódź University of Technology under the supervision of Urban (cf. works [46,47,48,49,50,51]). The experimental investigations conducted at Łódź University of Technology cover a wide range of issues regarding the punching shear of flat slabs made of concrete with sintered aggregate [46,47,48,49,50,51].

The ITB Laboratory conducted research on various mechanical properties of lightweight concretes with sintered aggregate [52,53], which concerned two types of concrete mixtures, due to equipment limitations. There are many extensive publications on concretes with lightweight aggregates, including a monograph [54]. The monograph presents in detail the properties of aggregates and concretes based on ash aggregates and lightweight concretes. Around the world, these materials are appreciated by both scientists and designers.

For this purpose, in the ITB Laboratory of Building Structures, Geotechnics, and Concrete, independently of the conducted research on the short-term strength properties of the considered lightweight concrete, tests were carried out to determine the long-term properties of shrinkage and creep of concrete. A program was developed to test the strength parameters and mechanical properties of lightweight concrete with sintered aggregate with appropriately selected parameters of the concrete mix. The tests were planned for two types of concrete, assuming mixtures analogous to those used in the works cited above [9,13]. Such robust experimental results are very important for structural design. Knowledge of the discussed parameters is essential to the design of complex engineering structures in accordance with current standards and, in particular, for the adoption of proven values for the static calculation of prestressed lightweight concrete elements.

## 2. Materials and Methods—Research Program

### 2.1. Characteristics of the Aggregate Used for Lightweight Concrete Mixes

**Figure 1:**
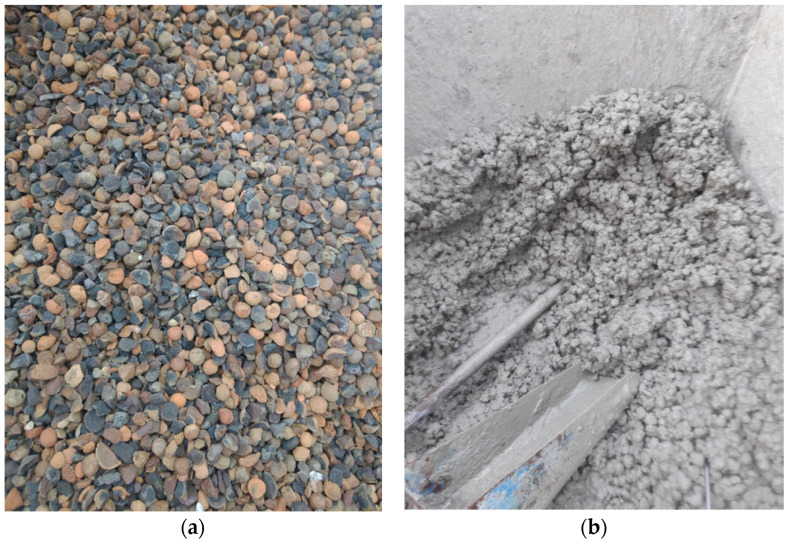
Lightweight sintered aggregate: (**a**) The aggregate. (**b**) Concrete mix with this aggregate.

**Figure 2:**
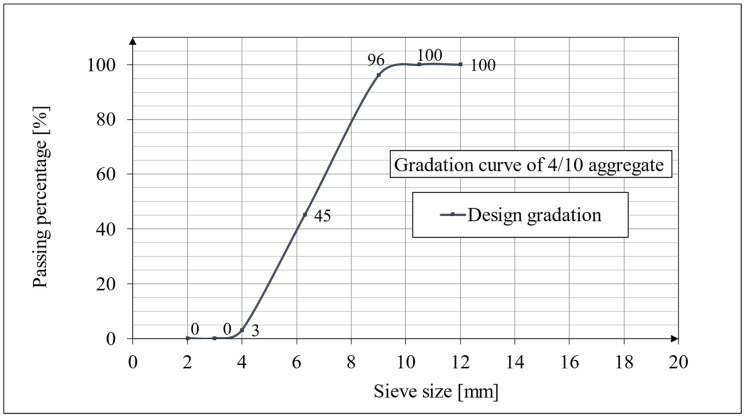
Gradation curve of 4/10 aggregate.

The scope of work covers the determination of the long-term mechanical properties of two lightweight concrete mixtures with a special ceramic, sintered aggregate (Certyd, Białystok, Poland). The sintered aggregate and the fresh concrete mix with this aggregate used in the work are shown in Figure 1. The aggregate used is a type of crushed aggregate with a fraction of 4/10. The manufacturer determined the percentage composition of the aggregate fraction by the screening method before it was allowed for testing, and the results were that 96% was a fraction smaller than 9 mm, 45% was smaller than 6.3 mm, and 3% was a fraction smaller than 4 mm. The dust content was 0.6%. In addition, the manufacturer provided the following information on the aggregate prepared for testing: bulk density: 654.9 kg/m^3^; crushing value: 6.5 Mpa; water absorption: 18.4% after 24 h; grain density: 1417 kg/m^3^. The design gradation curve of the 4/10 aggregate is shown in Figure 2.

### 2.2. Materials for Concrete Mixes

Two concrete mixtures with water/cement ratios (W/C) of 0.4 and 0.5, respectively, were made using the ingredients adopted in accordance with Table 3 (see below). The mixtures were prepared in the same way as in [8,9,13] (see Table 3 [13]), and the compositions of both mixtures were developed based on the recommendations for LSA [9]. The main difference is the aggregate fraction of 4/10, because the fraction 4/12 was no longer available. The main objective of the tests was to determine the compressive and tensile strength, secant modulus of elasticity, and creep and shrinkage strain of LC1 and LC2 concretes for comparative purposes, based on already tested concrete mixtures [8,9,13].

### 2.3. Methods of Tests and Corresponding Samples

The LWAC was prepared in a concrete plant by using a specialized mixer (manufactured by Liebherr-Mischtechnik GmbH, Bad Schussenried, Germany), which guarantees the homogeneity of the mixture. The features of fresh mixes were tested in accordance with the relevant standards [55,56,57,58,59]. Two sample preparation methods were adopted: cylindrical samples were collected from core drillings made in an LWAC block, while cubic and prismatic samples were cast in molds. Tests on the axial tensile strength and creep of LWAC (due to the lack of standards or difficulties in their implementation) are rarely performed, and so far, there is a lack of reliable test results in this area. Therefore, ITB Instruction No. 194/98, among others, was used for this purpose [60]. Cylindrical samples were taken from core drillings with a nominal diameter of 94 mm. The samples were prepared for testing the following mechanical properties of lightweight concrete:

- Secant modulus of elasticity in accordance with [61] (68 cylindrical samples);
- Compressive strength according to [62] (50 cylindrical and 36 cubical samples);
- Axial tensile strength according to [60] (50 cylindrical samples);
- Tensile splitting strength according to [63] (36 cubical samples);
- Flexural strength according to [64] (36 prismatic samples);
- Amsler shrinkage tests [65] (6 prismatic samples);
- Shrinkage tests according to [66] (6 cylindrical samples);
- Creep tests according to [60] (6 cylindrical samples).

When designing prestressed structures [67], it is important to adopt proven values, so research in the above-mentioned area is essential.

The test samples and LWAC block were formed according to EN 12390-1 [68] and EN 12390-2 [69]. After forming, the samples and concrete block were conditioned at a temperature of 20 ± 2 °C and a humidity > 95% for 28 days. After 28 days of maturation, the samples were matured at a temperature of 20 ± 2 °C under air-drying conditions with humidity > 50%.

For the remaining tests, the samples were prepared in accordance with the relevant standards as described above.

### 2.4. Types of Testing Machines

**Figure 3:**
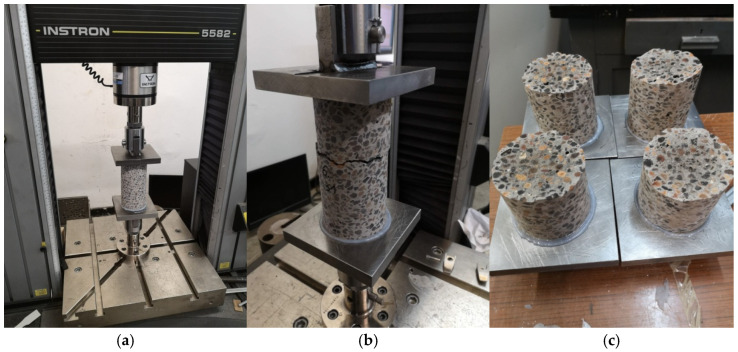
The axial tensile strength test: (**a**) The device used to test the axial tensile strength. (**b**) The broken sample after the test. (**c**) The forms of fracture of the samples.

**Figure 4:**
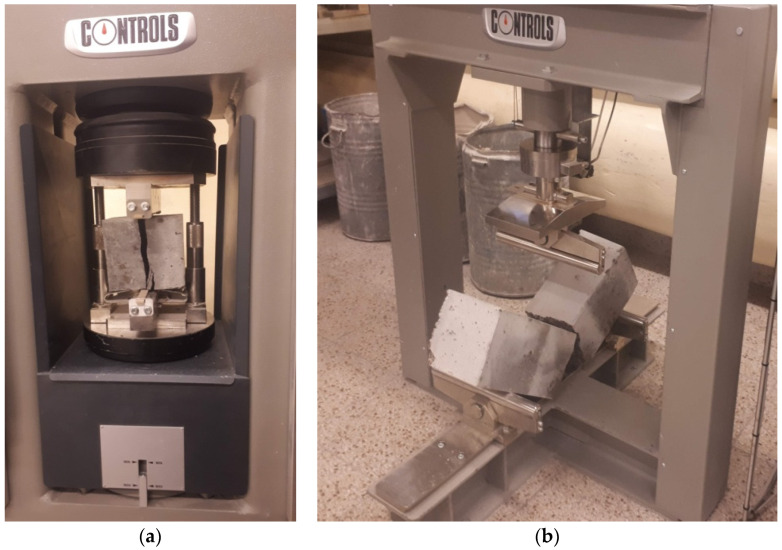
The tensile strength tests: (**a**) The device for testing tensile strength at splitting with the broken test specimen. (**b**) The device for testing bending strength with the broken test specimen.

**Figure 5:**
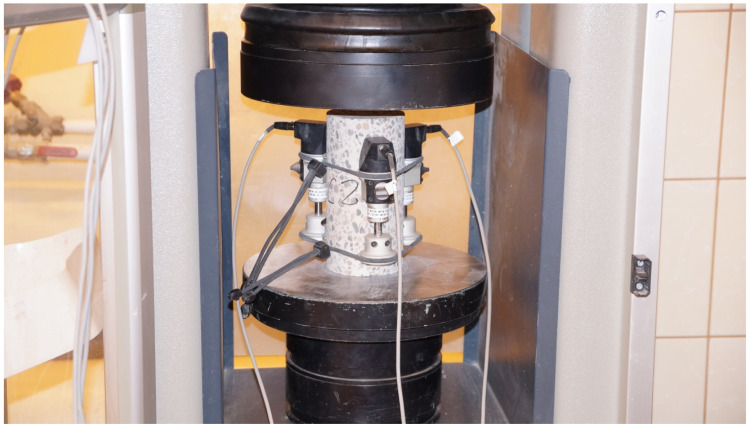
The device used for testing the modulus of elasticity.

**Figure 6:**
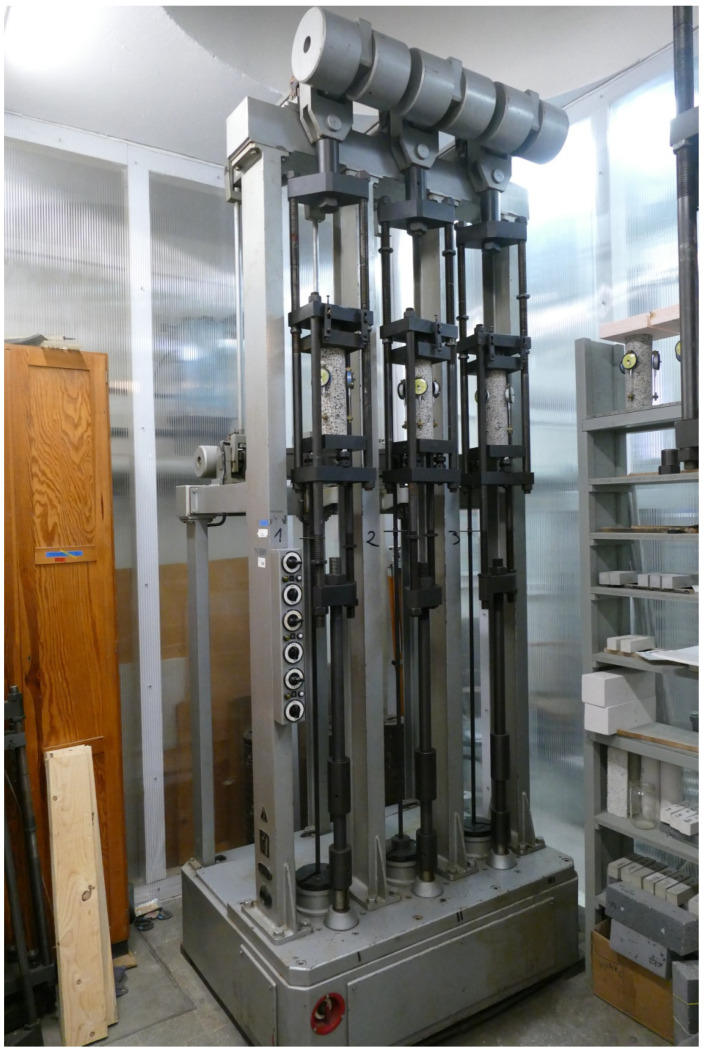
The creep-testing machine.

The basic devices used to test the mechanical properties of the considered lightweight concrete are presented below. The device used for the axial tensile strength tests, the sample after the test, and the fracture forms of the samples are shown in Figure 3a, Figure 3b, and Figure 3c, respectively. Figure 4a shows the apparatus for testing tensile strength at splitting and the sample split as a result of the test. The bending strength testing equipment and the sample broken as a result of the test is presented in Figure 4b. The device for testing the modulus of elasticity is shown in Figure 5. The last one is the creep-testing machine shown in Figure 6. The tests were carried out in accordance with the requirements of the relevant standards [55,56,57,58,59,61,62,63,64,65,66] and ITB Instruction No. 194/88 [60]. The testing equipment is in the first class of accuracy.

Creep and shrinkage strain tests were carried out in a special cabin while maintaining constant temperature and humidity.

### 2.5. General Schedule for Long-Term Studies

According to the experimental program, strength tests were performed after 7 and 14 days; then, after 28, 60, and 120 days; and finally, after 300 days. The shrinkage tests were carried out by the Amsler method at intervals consistent with the reference document [65], until the results stabilized). Only the secant modulus of elasticity and shrinkage development in accordance with the new standard [66] were studied for a longer period—for the purpose of creep strain testing. The entire creep deformation tests were carried out on six stands in creep-testing machines for 1050 days for LC1 concrete and 1044 days for LC2 concrete; however, in the present work, only the first loading and unloading are described. In addition, shrinkage was tested according to EN 12390-16 [66] in the same period of time. The conducted tests allowed us to determine the above-mentioned short-term and long-term parameters of concrete samples with lightweight sintered aggregate.

## 3. Results

### 3.1. Test Results of Concrete Mixture Properties

Two concrete mixes, LC1 and LC2, adopted in a similar manner as in [8,13] were tested on the basis of the relevant standards. Table 3 (given above) shows the recipe for the two types of concrete mixes used. The characteristics of the fresh mixes are presented in Table 4. Average consistency by the cone fall method (slump test) was tested according to EN 12350-2 [55], the consistency class according to EN 206 [56], the average air content according to EN 12350-7 [57], and the fresh concrete mix density according to EN 12390-6 [58].

After 28 days of curing, the concrete density values were determined to be 1766 kg/m^3^ for the dried concrete marked LC1 and 1777 kg/m^3^ for the concrete marked LC2 according to [59].

### 3.2. Test Results of Strength Properties of Lightweight Aggregate Concrete

#### 3.2.1. Test Results of Compressive Strength

**Figure 7:**
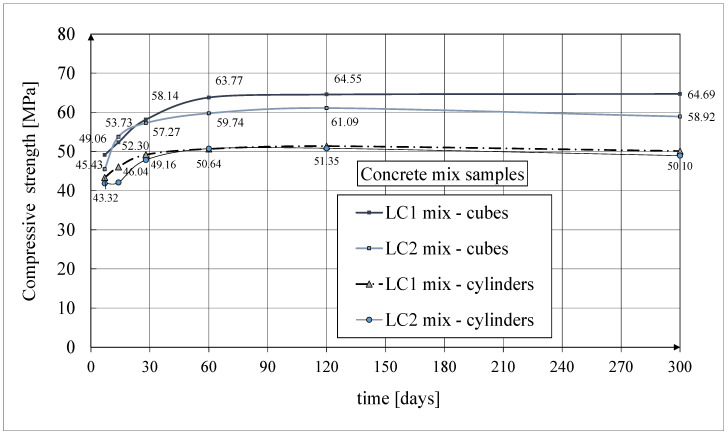
The compressive strength test results for cubical and cylindrical samples.

The test results of the compressive strength of cubical and cylindrical samples are shown in Figure 7. After 7 days from the preparation of the samples, the LC1 and LC2 concrete mixes obtained the LC 35/38 class according to [67] (see Figure 7). The numerical labels in the graphs refer to the cube strength and, in addition, in the case of the cylindrical strength, only to the results obtained from concrete samples for the LC1 mix due to overlapping graphs. At 28 days, these mixes achieved two following classes, finally reaching the LC 45/50 class according to [67] with the D1.8 density class.

The results of these tests up to 60 days of concrete age were previously published in the works by Rogowska and Lewiński [52,53]. Tests of concrete cube compressive strength were carried out every time for three typical samples with dimensions of 150 × 150 × 150 mm in accordance with the standard [62]. Samples (drilled from a lightweight concrete block) with a nominal diameter of 94 mm and a height of 190 mm were used to test the concrete’s cylindrical compressive strength. The graphs given in the above figure show the average strength test results obtained each time from 3 cylindrical samples, except for the concrete tests after 28 days from casting, which show the average results from 10 samples. In the case of the cylindrical compressive strength of the LC1 and LC2 concrete samples 28 days after their production, the mean strength values were 49.2 MPa and 47.8 MPa, respectively; the standard deviations were 2.49 MPa and 1.21 MPa, respectively; and the coefficients of variation were 5.07% and 2.53%, respectively. These results indicate very good homogeneity of the LC1 and LC2 concretes. In the case of the cube compressive strength obtained from three concrete samples for LC1 and LC2 28 days after their production, their average strength values were 58.3 MPa and 57.6 MPa, respectively; the standard deviations were 0.48 MPa and 0.87 MPa, respectively; and the coefficients of variation were 0.81% and 1.50%, respectively. Similarly, these values indicate very good homogeneity of the LC1 and LC2 concretes.

#### 3.2.2. Test Results of Tensile Splitting Strength

**Figure 8:**
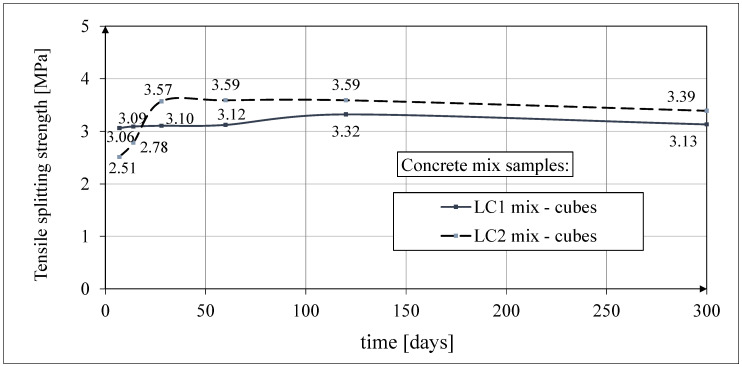
The tensile splitting strength results.

The splitting strength results for the LC1 mix over 60 days were fairly constant and these results for the LC2 mix increased by more than 40% between days 7 and 28. Tests of concrete tensile splitting strength were carried out each time on three typical cubic samples with dimensions of 150 × 150 × 150 mm in accordance with the standard [63]. The results of these tests up to 60 days of concrete age were previously published in the paper [52]. The test results of the tensile splitting strength of test specimens are given in Figure 8. The average values of the tensile strength of LC1 and LC2 concrete cube samples in the splitting test 28 days after sample production were 3.10 MPa and 3.57 MPa, respectively, with standard deviations of 0.18 MPa and 0.21 MPa, respectively.

#### 3.2.3. Test Results of Flexural Strength

**Figure 9:**
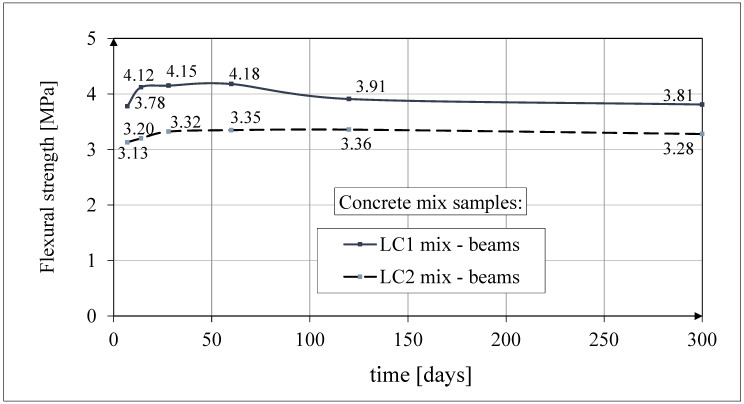
The comparison of test results of the flexural strength.

The results of the concrete flexural strength tests are shown in Figure 9. The tests of flexural strength were carried out each time for both concrete mixes on three prismatic samples with dimensions 550 × 150 × 150 mm in accordance with the standard [64], and the spacing of the support rollers was 450 mm.

The flexural strength results for the LC2 concrete over 60 days were fairly constant, while those for the LC1 concrete increased by more than 10% between 7 and 28 days. The results of these tests up to 60 days of concrete age were published earlier in the paper [52]. The average values of the tensile strength of the LC1 and LC2 concrete samples in the flexural test 28 days after sample production were 4.15 MPa and 3.32 MPa, respectively, with standard deviations of 0.21 MPa in both cases.

#### 3.2.4. Test Results of Axial Tensile Strength

**Figure 10:**
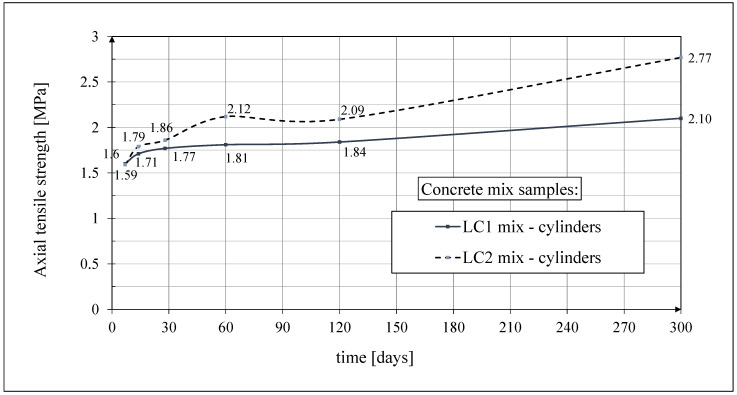
The test results of the axial tensile strength.

The test results of the development of the axial tensile strength of the LC1 and LC2 concretes according to ITB Instruction No. 194 [60] 14, 28, 60, 120, and 300 days after sample production are given in Figure 10. The axial tensile strength tests were performed on 3 cylindrical samples each time, except for the tests 28 days after casting, which represent average results from 10 samples. Samples with a diameter of *d* = 94 mm and a height of *h* = 190 mm were used, selecting samples with the same cross-section as those intended for the creep tests (and accompanying shrinkage tests). Seven days after sample preparation, the LC1 and LC2 concretes reached an average axial tensile strength of approximately 1.6 MPa (see Figure 10). After 28 days, the strength increased by more than 10%. The results of these tests up to 60 days of concrete age were published in the works [52,53]. In both cases, the axial tensile strength of LC1 and LC2 cylindrical concrete samples was determined after 28 days from sample production. The average values of this strength were 1.77 MPa and 1.86 MPa, respectively, and the standard deviations were 0.57 MPa and 0.34 MPa, respectively.

The coefficients of variation of the tensile strength in the splitting test of LC1 and LC2 cubic concrete samples 28 days after making the samples were 5.84% and 5.78%, respectively. The coefficients of variation of the average values of the tensile strength in the bending test of LC1 and LC2 prismatic concrete samples 28 days after making the samples were 5.01% and 6.28%, respectively. This means that from the point of view of the tensile strength of this concrete under splitting and bending, the homogeneity of this feature of both the LC1 and LC2 concretes is very good. However, the coefficients of variation of the axial tensile strength of the LC1 and LC2 cylindrical concrete samples after 28 days from sample preparation were 31.94% and 18.21%, respectively, which means that in contrast to the tensile strength under splitting and bending of the concrete under consideration, the inhomogeneity of this feature of both the LC1 and LC2 concretes is very large, and the axial tensile strength is a weak point of this concrete, so it is quite brittle.

This differentiation in the tensile strength of samples depending on the method of testing the samples requires some comment. The axial tensile strength test seems to be the most objective, but it has a significant drawback. While the method of testing the tensile strength under splitting and bending does not change its essence during loading, in the case of the axial tensile strength test, unintended eccentricity may occur due to the inhomogeneity of concrete in the cross-section, which in turn means that the sample, assumed to be under axial tension, is in fact subjected to a tensile force and a bending moment of unknown value, which in turn leads to a series of results with a fairly large and varied standard deviation. This does not mean that there is only one correct way to test tensile strength, because different mechanisms of failure in tension occur in different stress states. For example, axial tension may occur when a wall made of the concrete under consideration connects two similarly constructed floors, the lower one of which is more heavily loaded than the upper one; then, the crack will be horizontal. Tension under splitting may occur below the edge of the beam, where it rests on the lightweight concrete wall, and tension under bending may occur, e.g., in a floor slab made of the tested concrete. In all three cases, cracks will appear at different stress levels, and this is because the appearance of these cracks is not actually determined by stress but by the strain exceeding a certain permissible limit.

#### 3.2.5. Estimation of Measurement Uncertainty

The uncertainty of measurement results is closely related to any test result. Although error analysis has long been a part of metrology, the concept of uncertainty as a feature expressed numerically is relatively new. The results of strength tests of LC1 and LC2 concrete samples with lightweight sintered aggregate for concrete ages *t* = 7, 14, 28, 60, 120, and 300 days were developed together with the expanded measurement uncertainty (*U*). The calculation of this uncertainty was based on the normal distribution, an expansion probability of about 95%, and a coverage factor *k* = 2. The standard uncertainties were determined using corrections related only to the accuracy of the measuring devices used.

The determined expanded uncertainty of the measurement (*U*) in percentage terms did not exceed the following values:

- Cube compressive strength: 1.28% for the LC1 concrete and 1.24% for the LC2 concrete;
- Axial tensile strength: 1.35% for the LC1 concrete and 1.84% for the LC2 concrete.

As can be easily seen, the uncertainties obtained in the strength test results of both compressive strength and axial tensile strength are very small and smaller than the coefficients of variation of the particular strength properties.

### 3.3. Results of Research and Analysis of Long-Term Properties of Lightweight Aggregate Concrete

#### 3.3.1. Results of Research and Analysis of Secant Modulus of Elasticity

- Test results of secant modulus of elasticity

**Figure 11:**
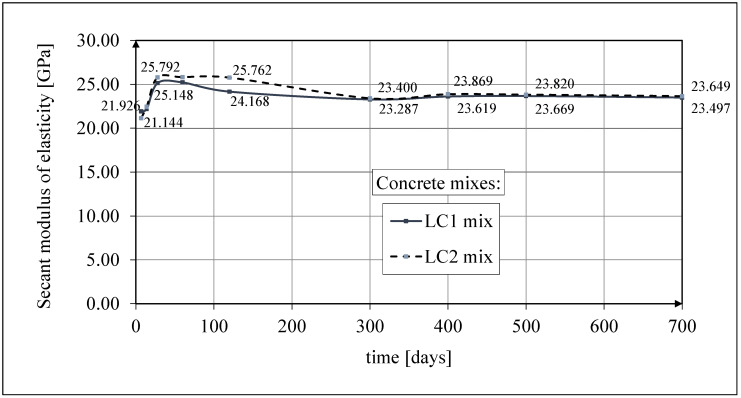
The test results of the secant modulus of elasticity.

The results of the tests of the secant modulus of elasticity of concrete are shown in Figure 11. The results of the tests up to 60 days of concrete age were published in the works [52,53]. The tests of the secant modulus of elasticity of concrete in compression were carried out according to the standard [61] on samples with diameter *d* = 94 mm and height *h* = 190 mm, selecting the same cross-section of samples for testing as those intended for creep tests (and accompanying shrinkage tests). The graphs given in Figure 11 show the average test results of the secant elasticity modulus of concrete obtained each time for 3 cylindrical samples, except for the concrete tests 28 days after casting, which show the average results for 10 samples. The values of the secant modulus of elasticity of LC1 and LC2 concrete samples 28 days after their production were determined in both cases based on the testing of 10 cylindrical samples, with the average values of these moduli being 25,150 MPa and 25,790 MPa, respectively; the standard deviations being 1135 MPa and 601 MPa, respectively; and the coefficients of variation of the modulus of elasticity being 4.51% and 2.33%, respectively. These results indicate very good homogeneity of the concrete in this range, for both LC1 and LC2 concrete samples.

The test results for the two concrete mixes, LC1 and LC2, showed a similar increase over 60 days. The secant modulus of elasticity increased by approximately 13% between 7 and 28 days. The course of the changes in the modulus of elasticity of the tested concrete with lightweight sintered aggregate, illustrated in Figure 11, is not obvious. From the previously cited literature sources, it results that the values of the secant modulus of elasticity of concrete are inversely proportional to the value of drying shrinkage. The curing of concrete was completed after 28 days. The purpose of curing concrete is, among others, to reduce the amount of drying shrinkage. However, drying shrinkage is different in the case of concrete with porous aggregate compared with plain concrete. The research described below shows that after 28 days, the shrinkage strain had not yet reached half their final value. Completing the curing process may coincide with water drying in aggregate pores due to moisture migration in concrete. In this case, there will be an increase in drying shrinkage in the entire volume of concrete after the end of the curing process. Increased shrinkage stresses (in contrast to plain concrete) are not compensated by the increase in the strength of the cement matrix due to its accelerated drying in the zones of contact with the porous aggregate. This may initiate the formation of micro-cracks, which do not significantly impact the compressive strength of lightweight concrete, but after the curing is completed, they may reduce the tensile strength and the value of the elasticity modulus of the concrete under consideration. The effect of the initial wetting of lightweight aggregate on the tensile strength of concrete and its long-term properties under tension was the subject of tests in many papers, e.g., in [28,70,71], and so was the effect of concrete curing time, e.g., in [72]; the effect of lightweight aggregate impregnation was analyzed in [3].

- Measurement uncertainty of modulus of elasticity

The determined expanded uncertainty of the measurement (*U*) of the secant modulus of elasticity given in percentage terms did not exceed the values of 2.03% for LC1 concrete and 1.92% for LC2 concrete. As can be easily seen, the obtained measurement uncertainty of the test results of the modulus of elasticity is very small and smaller than the coefficients of variation of the particular modules.

- Analytical results of secant modulus of elasticity models

The secant modulus of elasticity can be represented in relation to the compressive strength of concrete by the formula following the CEB-FIP Recommendations [73] and in relation to time as by the formula given in Eurocode 2 [67]. The mutual empirical relation of the secant modulus of elasticity of concrete in relation to the compressive strength is proposed herein by analogy with the CEB-FIP Recommendations [73] by means of the following empirical relationship:

*E_c_* = *β* (*f_c_*)^1/2^. (1)

Based on our research, the following experimental constant was determined: *β* = 3.0. *f_c_* is expressed in MPa, while *E_c_* is expressed in GPa.

**Figure 12:**
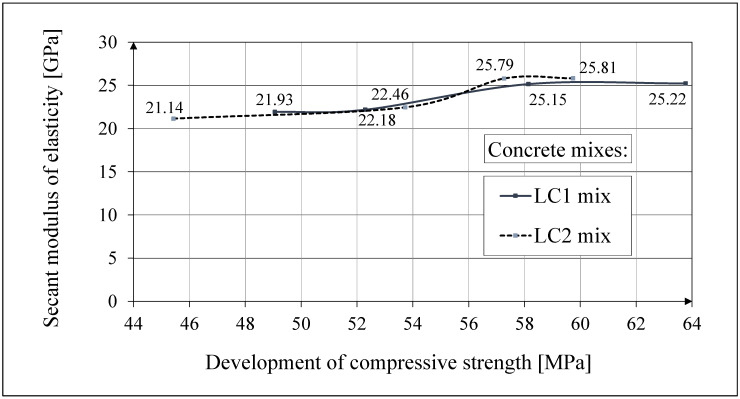
The test results of the secant modulus of elasticity in relation to compressive strength.

**Figure 13:**
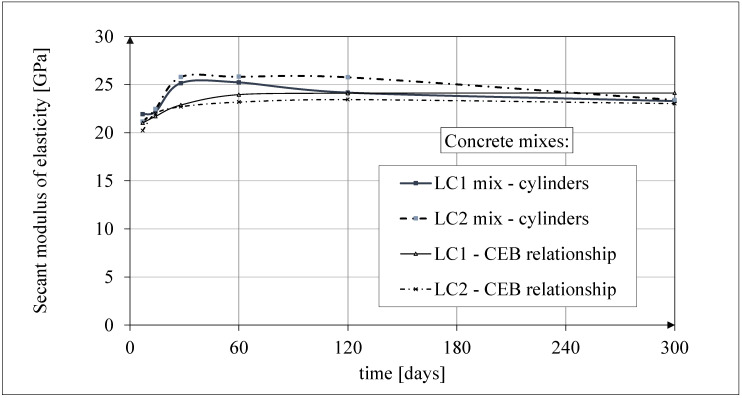
The comparison of the test results of the secant elasticity modulus with the analytical results.

The comparison of the test results of the secant modulus of elasticity of concrete in relation to the compressive strength of concrete samples made of two concrete mixes, LC1 and LC2, is shown in Figure 12. The results of this comparison were published earlier in the paper [52]. The comparisons of the test results of the secant modulus of elasticity of concrete as a function of time [in days] and the analytical results according to the CEB-FIP Recommendations [73] for the concrete samples made of two concrete mixtures, LC1 and LC2, and their mutual relationship are presented in Figure 13. The results of these comparisons up to 60 days of concrete age were published earlier in the article [52].

**Figure 14:**
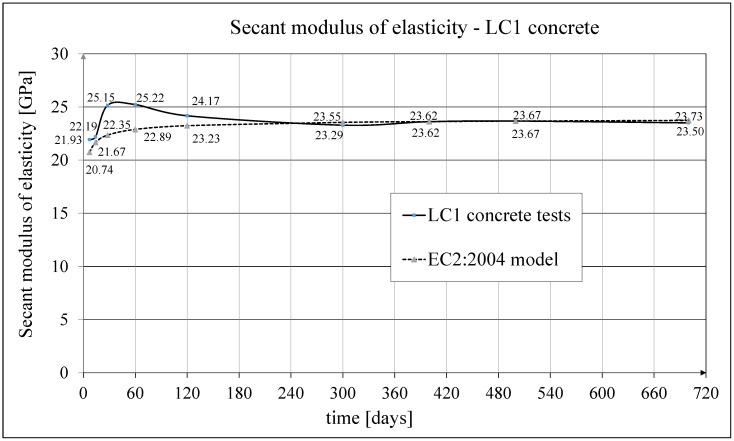
The comparison of the test results of the secant elasticity modulus with the EC2:2004 model for LC1 concrete.

**Figure 15:**
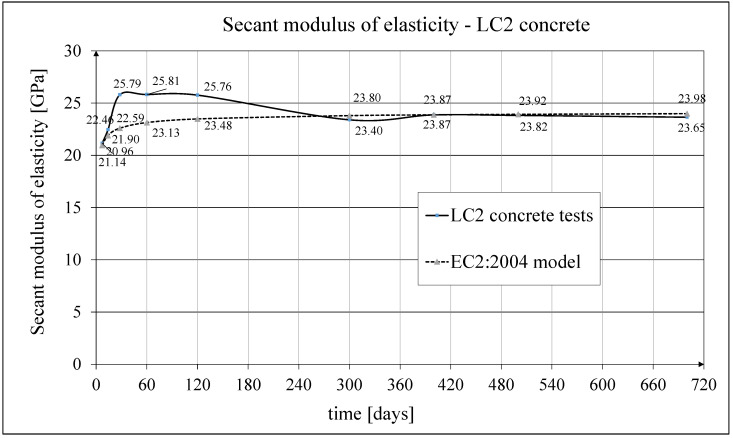
The comparison of the test results of the secant elasticity modulus with the EC2:2004 model for LC2 concrete.

**Figure 16:**
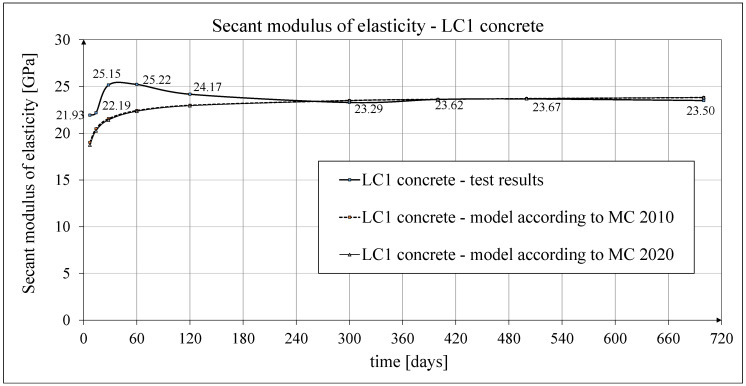
Development of secant modulus of elasticity of LC1 concrete according to tests and according to MC 2010 and MC 2020.

**Figure 17:**
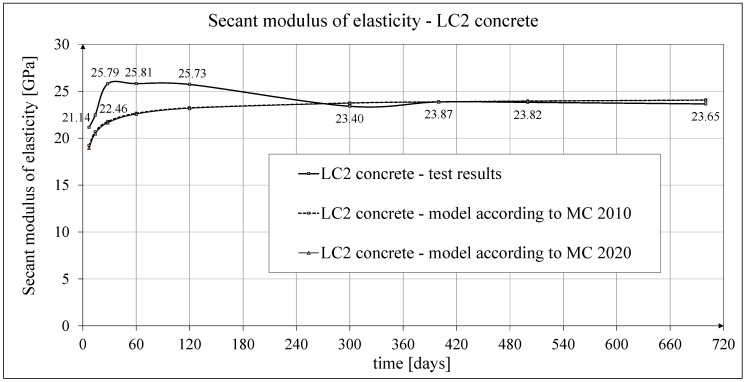
Development of secant modulus of elasticity of LC2 concrete according to tests and according to MC 2010 and MC 2020.

The development of the secant elasticity modulus of concrete in time for the samples made of two concrete mixtures, LC1 and LC2, as well as the analytical results of this parameter obtained by the formula contained in EC2:2004 [67], are shown in Figure 14 and Figure 15, respectively. The comparisons of the secant modulus of elasticity for LC1 and LC2 concrete samples according to tests and models of pre-standards MC 2010 [74] and MC 2020 [75] (the same model as in EC2:2023 [76]), are presented in Figure 16 and Figure 17, respectively.

#### 3.3.2. Results of Research and Analysis of Shrinkage Strain

- Test results of shrinkage strain

**Figure 18:**
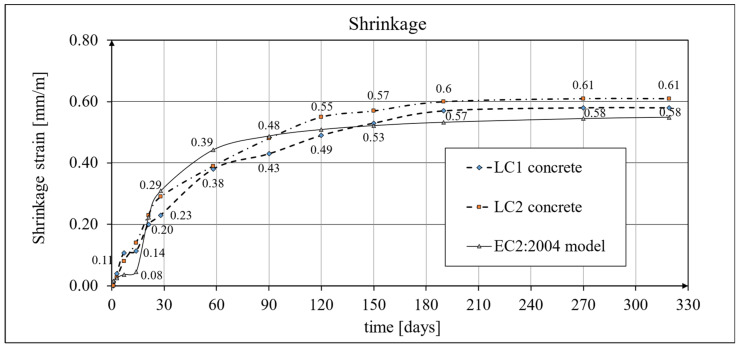
The development of shrinkage according to the Amsler tests and the EC2:2004 model.

**Figure 19:**
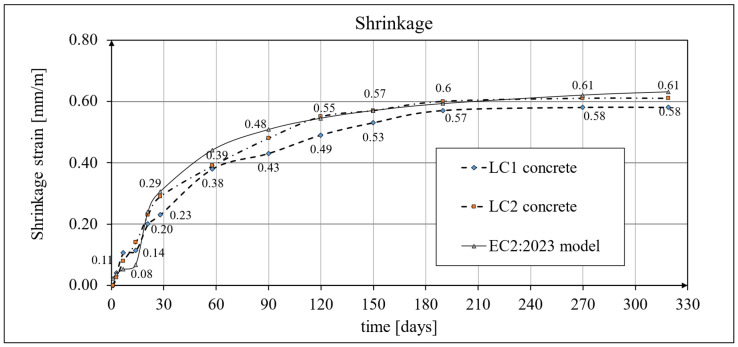
The development of shrinkage according to the Amsler tests and the EC2:2023 model.

Two methods of measuring shrinkage strain were adopted: tests using the standard Amsler method [65], which were carried out on prismatic samples with dimensions of 100 × 100 × 500 mm, and tests conducted in accordance with the current EN 12390-16 standard [66] on cylindrical samples with diameter *d* = 94 mm and height *h* = 3*d* = 282 mm, selecting the same geometry of the samples as those intended for creep tests, in accordance with ITB Instruction No. 194/98 [60]. In both tests, the strain was measured in three samples made of the LC1 and LC2 concrete mixtures (a total of 12 specimens). The results of the tests conducted using the Amsler method [65] included readings initially taken at intervals of several days and then weekly, monthly, and longer intervals. The entire research process lasted 325 days from the time the samples were made. The results of the shrinkage development tests of LC1 and LC2 concrete samples conducted using the Amsler method [65] are shown in Figure 18 and Figure 19.

**Figure 20:**
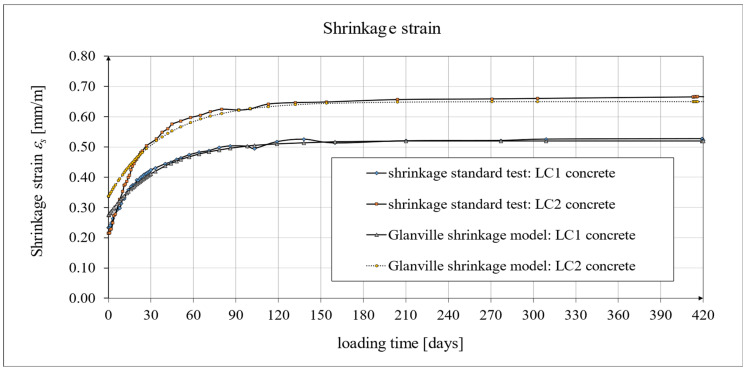
The development of shrinkage according to the standard tests [66] and the Glanville model [77].

**Figure 21:**
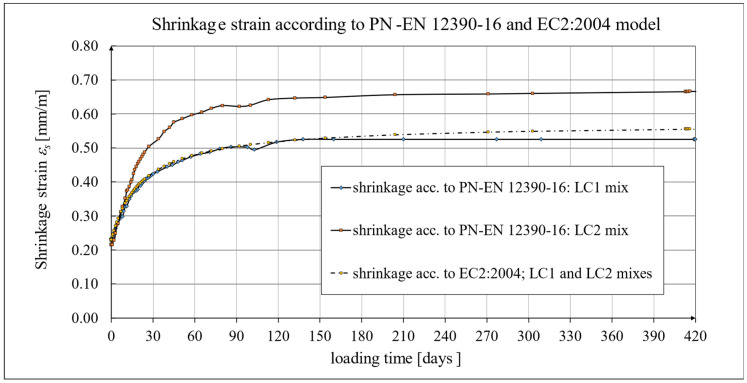
The development of shrinkage according to the EN 12390-16 standard tests [66] and the EC2:2004 standard model [67].

**Figure 22:**
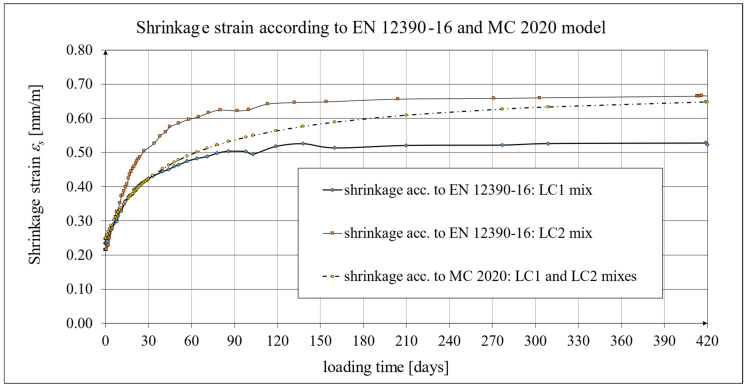
The development of shrinkage according to the EN 12390-16 standard tests [66] and the EC2:2023 standard model [76], as well as MC 2010 [74] and MC 2020 [75].

Creep and shrinkage measurements were carried out on samples stored in a special laboratory cabin, where temperature and humidity were monitored and kept constant. Concrete samples marked LC1 and LC2 showed similar increases in shrinkage. However, the LC1 mix showed very little shrinkage between days 7 and 14. Consistent results were obtained for both lightweight concretes at 58 days, but the shrinkage of the LC1 concrete was ultimately found to be slightly lower. The values of the shrinkage strain stabilized after 270 days from the preparation of samples from both concrete mixtures, with an average value of 0.58 mm/m and a standard deviation of 0.020 mm/m for LC1 concrete and an average value of 0.61 mm/m and a standard deviation of 0.031 mm/m for LC2 concrete, and the coefficients of variation of the shrinkage strain were 3.45% and 4.98%, respectively. The test results up to the 58th day of concrete age were previously published in the papers [52,53]. The tests carried out in accordance with the EN 12390-16 [66] standard on cylindrical specimens were of auxiliary character in relation to the creep tests and were started after 28 days of concrete setting in the block used for taking the samples, and the increases in shrinkage strain were measured only after taking the samples. During this time, the specimens used for measuring elastic and total strain were loaded. The creep strain was determined by subtracting the elastic and shrinkage strain from the total strain. In order to prepare the shrinkage strain diagrams of the specimens tested according to the EN 12390-16 [66] standard, it was necessary to add the initial values of the shrinkage strain (in both cases, 0.23 mm/m was assumed based on the Amsler test) to the final shrinkage strain increments occurring during the creep tests with average values of 0.29 mm/m in the case of LC1 concrete and 0.45 mm/m in the case of LC2 concrete. Therefore, the strain values in these graphs (Figure 20, Figure 21 and Figure 22) are approximate. Under such assumptions, for the LC1 concrete at age *t* = 419 days, the approximate mean shrinkage strain was obtained as equal to 0.52 mm/m with a standard deviation of 0.044 mm/m, and for the LC2 concrete at age *t* = 413 days, the approximate mean shrinkage strain was obtained as equal to 0.68 mm/m with a standard deviation of 0.067 mm/m.

The expanded measurement uncertainty *U* (defined above) of the shrinkage strain determined by the Amsler method (*ε_sA_*) for LC1 lightweight concrete at the ages of 270 and 325 days (*ε_sA_* = 0.58 mm/m) and the shrinkage of LC2 lightweight concrete at the ages of 270 and 319 days (*ε_sA_* = 0.61 mm/m—see Figure 18) is *U* = 0.03 mm/m.

The expanded measurement uncertainties *U* of the shrinkage strain determined in this way in percentage terms were 5.2% for LC1 lightweight concrete at the ages of 270 and 325 days and 4.9% for LC2 lightweight concrete at the ages of 270 and 319 days.

- Analytical results of shrinkage models

The results of shrinkage strain tests can be approximated using the Glanville model [77], in which the theoretical curves were adopted according to the following relationship:

*ε* (*t*) = *ε*_0_[1 − exp(−*s t*)]. (2)

A comparison of the concrete shrinkage development as a result of tests made according to the EN 12390-16 standard [66] and based on the Glanville model [77] (see Equation (2)) during creep strain tests is shown in Figure 20, where *s* = 0.027 and *ε*_0_ = 0.00052 were adopted for LC1 concrete and *s* = 0.026 and *ε*_0_ = 0.00065 for LC2 concrete to determine the additional shrinkage (*ε_as_*) after 28 days of concrete binding.

The description of shrinkage development was adopted according to the EC2:2004 model [67] and, for comparison, according to the EC2:2004 model [67]. The results of the shrinkage development tests of LC1 and LC2 concrete samples conducted using the Amsler method [65] and the shrinkage values determined for comparison according to the EC2:2004 model [67] are presented in Figure 18. The shrinkage strain of the samples of both concretes tested and determined according to the EC2:2023 model [76] is shown in Figure 19. The obtained results in terms of shrinkage strain are much higher than in the cited paper [9].

The test results were compared with the calculation results based on the models according to the EC2:2004 standard [67] and to the EC2:2023 standard [76] taking into account the characteristics of lightweight concrete class LC 45/50 according to [67], the concrete age at the beginning of drying of *t_s_* = 14 days, and the drying shrinkage factor *η* = 1.2 in both models. The test results for the two concrete mixtures, LC1 and LC2, conducted according to the EN 12390-16 standard [66], were compared to the shrinkage values determined according to the EC2:2004 model [67] (Figure 21) and the standard EC2:2023 model [76], and thus MC 2010 [74] and MC 2020 [75] (Figure 22), assuming in both models the concrete age at the beginning of drying of *t_s_* = 21 days and the drying shrinkage factor *η* = 1.2. In the case of tests using the Amsler method, the samples were stored under constant temperature and humidity conditions but without humidification. In the case of tests according to the EN 12390-16 standard [66], the concrete blocks from the LC1 and LC2 mixtures were stored under the same conditions, except that the samples were taken only 28 days after the production of these concrete blocks, which was less conducive to the drying of the samples. Therefore, in the tests conducted in accordance with EN 12390-16 [66], a later age of concrete at the beginning of drying was assumed. All shrinkage deformation tests using the standard method [66] were carried out in a special creep-testing cabin for 1050 days for LC1 concrete and 1044 days for LC2 concrete; however, after a period of approximately half a year from the commencement of the loading of the samples, the shrinkage stabilized, and further results after a period of 420 days are not presented in this paper.

#### 3.3.3. Results of Research and Analysis of Creep Strain

- Test results of creep strain

Creep measurements of the samples taken from the two concrete mixes, LC1 and LC2, were carried out in both cases on three samples with diameter *d* = 94 mm and height *h* = 3*d* = 282 mm in accordance with ITB Instruction No. 194/98 [60] (the same type of samples as used for shrinkage tests according to the standard method [66]). The lightweight concrete samples were 28 days old at the first loading. The strain was measured for both LC1 and LC2 concretes (see Figure 6) assuming the long-term loading program; however, in the present work only the first loading and unloading are described in detail:

- For concrete from the LC1 mix, there were three loading phases, which translated into a stress value of 15.55 MPa, and two unloading phases, which translated into a stress value of 1.56 MPa. The first loading phase lasted for the first 419 days, the first unloading phase lasted until day 572 after the first load application, and the loading–unloading process lasted for 1050 days.
- For concrete from the LC2 mix, there were three loading phases, which translated into a stress value of 16.96 MPa, and two unloading phases, which translated into a stress value of 1.70 MPa. The first loading phase lasted for the first 413 days, the first unloading phase lasted until day 566 after the first load application, and the loading–unloading process lasted for 1044 days.

**Figure 23:**
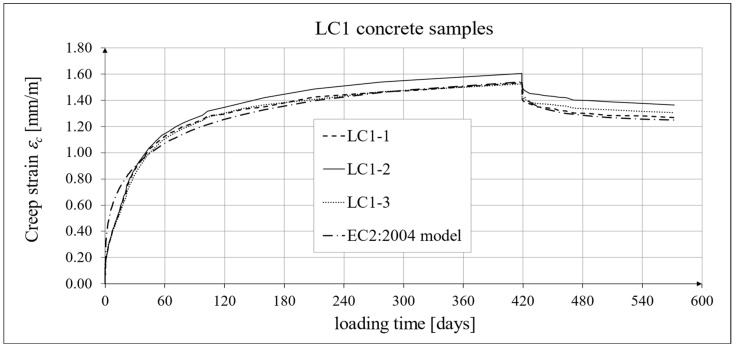
Creep strain of LC1 concrete for the first 572 days of loading and EN 1992-1-1:2004 [67] model predictions.

**Figure 24:**
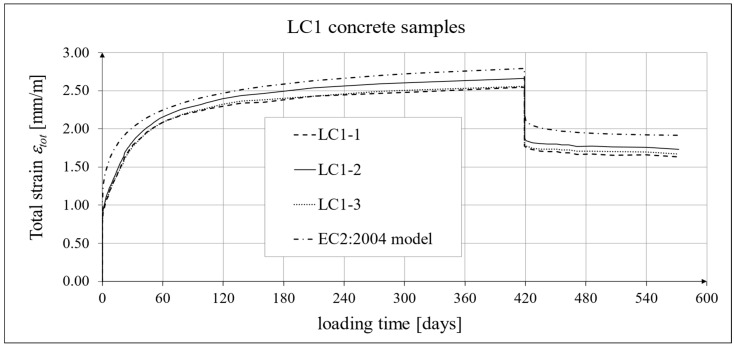
Total strain of LC1 concrete for the first 572 days of loading and EN 1992-1-1:2004 [67] model predictions.

**Figure 25:**
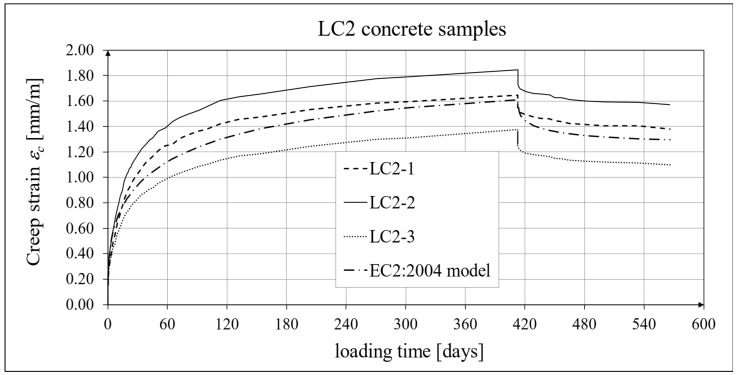
Creep strain of LC2 concrete for the first 566 days of loading and EN 1992-1-1:2004 [67] model predictions.

**Figure 26:**
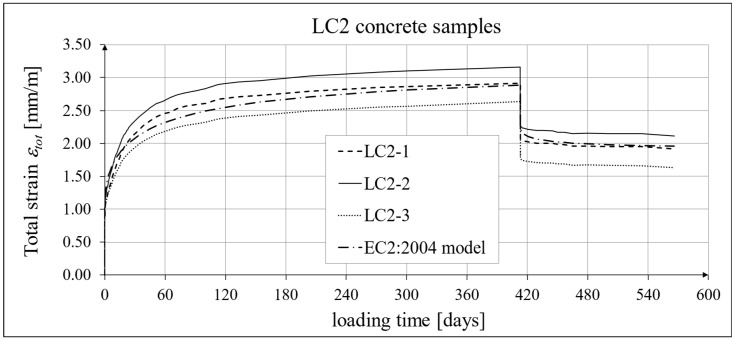
Total strain of LC2 concrete for the first 566 days of loading and EN 1992-1-1:2004 [67] model predictions.

The results of the creep strain for the LC1 concrete are shown in Table 5 for the loading time of *t* = 419 days, and the results of the creep strain for the LC2 concrete are shown in Table 6 for the loading time of *t* = 413 days. Creep tests were performed on three samples of LC1 concrete and three samples of LC2 concrete, which was due to equipment limitations in the laboratory. For this reason, the deformations of each sample were monitored individually, and the average values were determined only to determine the average creep coefficient obtained from three samples for both of the above concretes. The results concerning the creep strain and total strain of the LC1 concrete samples are shown in Figure 23 and Figure 24, respectively, where the first loading phase lasted for 419 days and the first unloading phase lasted until day 572 after the first load application. The results concerning the analogous strain of the LC2 concrete samples for the first loading phase lasting for 413 days and the first unloading phase lasting until day 566 after the first load application are shown in Figure 25 and Figure 26, respectively.

Theoretically, the creep coefficient, which is calculated as the ratio of creep strain to elastic strain, *φ* = *ε_c_*/*ε_e_*, is determined for *t* = ∞, but practically, for a period of not less than 1 year [60]. The average creep coefficient obtained from three samples of LC1 concrete was about 2.10 for the loading time of *t* = 419 days (which is consistent with the data given in Table 5), and in the case of LC2 concrete, it was about 1.90 for the loading time of *t* = 413 days (which is consistent with the data given in Table 6). The average creep coefficient obtained in the work [13] for both concretes was from about 0.59 to 0.60, which differs from both the results of these tests and the predictions of the code models [67,72,76,77]. The results for the LC2 concrete are characterized by a certain scatter, which is due to the unintentional inhomogeneity of the LC2 mix, which in turn results from the conditions of aggregate preparation before the concrete mix was made (outside the ITB laboratory).

- Measurement uncertainty of strain and statistical evaluation

The measurement uncertainty of the total (*ε_tot_*), elastic (*ε_e_*), and creep strain (*ε_c_*) of the LC1 lightweight concrete with sintered aggregate after the loading time of *t* = 419 days is given in Table 5, where *U*—expanded measurement uncertainty, stated as the combined standard measurement uncertainty multiplied by the coverage factor *k* = 2 such that the coverage probability corresponds to approximately 95%. The average value of the creep coefficient (*φ* = *ε_c_*/*ε_e_*) for the loading time of *t* = 419 days was 2.11, and the standard deviation was 0.037.

The measurement uncertainty of the total, elastic, and creep strain of the LC2 lightweight concrete with sintered aggregate after the loading time of *t* = 413 days is given in Table 6, where *U* and *k*—as above. The average value of the creep coefficient for the loading time of *t* = 413 days was 1.94, and the standard deviation was 0.214.

The determined expanded uncertainty of the measurement (*U*) in percentage terms did not exceed the following values:

- Total strain (*ε_tot_*): 2.75% for the LC1 lightweight concrete and 2.65% for the LC2 concrete;
- Elastic strain (*ε_e_*): 5.56% for the LC1 lightweight concrete and 4.94% for the LC2 concrete;
- Creep strain (*ε_c_*): 4.58% for the LC1 lightweight concrete and 5.07% for the LC2 concrete.

As can be seen, the measurement uncertainties obtained for the long-term strain test results are slightly higher than those obtained for the strength test results. This was due to the type of sensors used for long-term strain measurements; nevertheless, they are quite small compared with the dispersion of the long-term strain measurement results.

- Analytical results of creep models

Creep measurements were carried out on samples taken from two concrete mixes: LC1 and LC2 (see Figure 6). Creep tests were carried out on three samples of the LC1 concrete and three samples of the LC2 concrete. The charts included in the text show the results of the measurements of the creep strain only and the total strain of the samples. The values of the creep strain are defined as the difference between the total strain and the elastic and shrinkage strain (measured on cylindrical specimens by using the standard method [66]). Creep deformation analysis was also carried out based on seven creep models. However, the presented charts show the results obtained using the models given in standards EN 1992-1-1:2004 [67] and EN 1992-1-1:2023 [76], assuming the LC 45/50 concrete class, *RH* = 50%, and *h*_0_ = 47 mm, but with appropriate correction factors according to standards (*η_E_*, *ξ*_c_). The correction factors (*ξ*_c_) were determined in accordance with standard EN 1992-1-1:2023 [76] by minimizing the sum of squares of the differences between the model estimation and the experimental results. When comparing the results of the tests of the creep strain of the LC1 concrete with the calculation results according to the EC2:2004 standard [67] (at *η_E_* = 0.67, as above), in the case of the initial load, the resulting correction factor determined by the least square error method, amounting to *η_E_ ξ*_c_ = 1.28, was applied to the creep curve, while in the case of the LC2 concrete, the resulting factor calculated in the same way, *η_E_ ξ*_c_ = 1.24, was applied. This ensured the consistency of the results of the EC2:2004 model with the test results for the loading period from 0 to 419 days for LC1 concrete and, for LC2 concrete, with the test results from 0 to 413 days. However, in the case of creep deformations due to unloading occurring after a period of 419 and 413 days, for the two concretes, respectively, only a correction factor *η_E_* was applied, because in pre-standard MC 2020 [75] and in standard EN 1992-1-1:2023 [76], no other factors are specified for the unloading phase. A comparison of the creep strain of LC1 and LC2 concrete samples according to the tests and the model of the EC2:2004 standard [67] is shown in Figure 23 and Figure 25, respectively, and the comparison of the total strain of both concrete samples according to the tests and the considered model is shown in Figure 24 and Figure 26, respectively.

**Figure 27:**
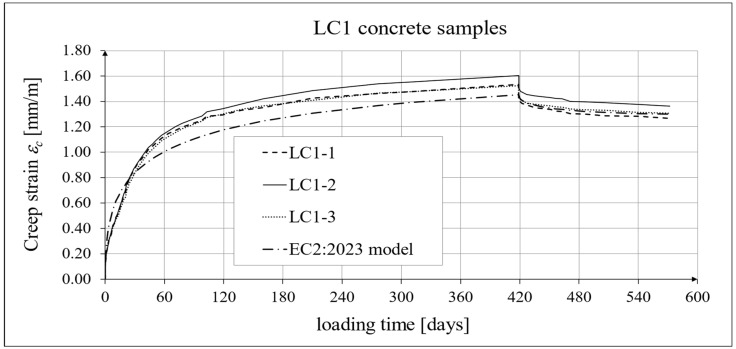
Creep strain of LC1 concrete for the first 572 days of loading and EN 1992-1-1:2023 model predictions.

**Figure 28:**
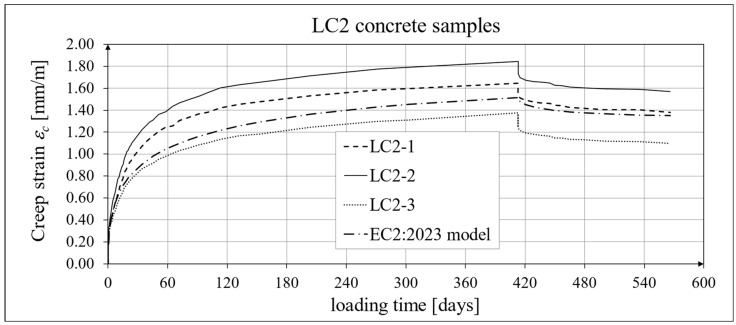
Creep strain of LC2 concrete for the first 566 days of loading and EN 1992-1-1:2023 model results.

**Figure 29:**
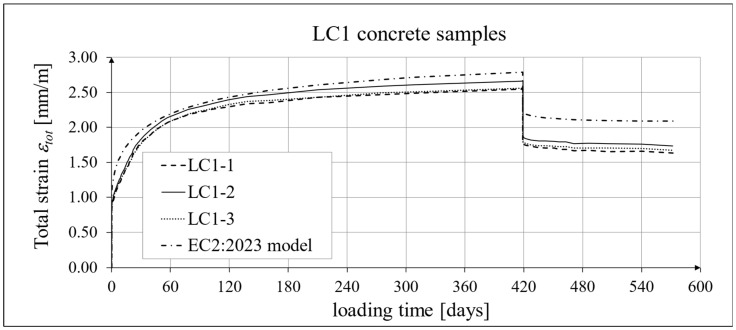
Total strain of LC1 concrete for the first 572 days of loading and EN 1992-1-1:2023 model predictions.

**Figure 30:**
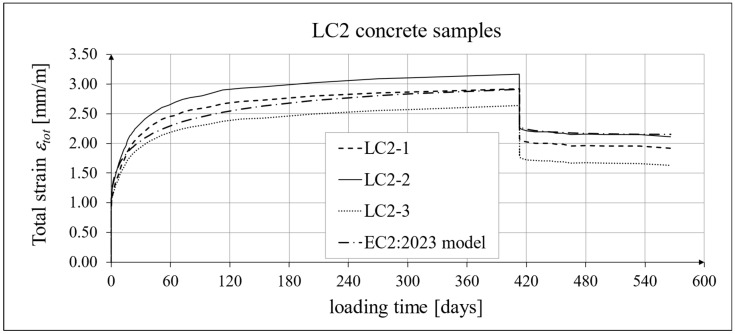
Total strain of LC2 concrete for the first 566 days of loading and EN 1992-1-1:2023 model results.

Similarly, in the case of comparing the results of the creep deformation tests of LC1 concrete with the results of MC 2020 [75] in the case of the creep curve for the initial load, the resulting correction factor determined by the method of least square errors *η_E_ ξ*_c_ = 1.28 was used, and in the case of LC2 concrete, we used the correction factor determined in the same way, *η_E_ ξ*_c_ = 1.24, which ensured the compliance of the results of MC 2020 with the test results for the load period from 0 to 419 days (LC1 concrete) or 413 days (LC2 concrete). In the case of unloading occurring after a period of 419 and 413 days for the two concretes, respectively, only the correction factor *η_E_* was applied, because pre-standard MC 2020 [75] does not specify other factors for the unloading phase. The comparison of the creep strain of LC1 and LC2 concrete samples according to the tests and the model of standard EN 1992-1-1:2023 [76] is presented in Figure 27 and Figure 28, respectively, and the comparison of the total strain of the samples of both concretes according to the tests and EC2:2023 model is presented in Figure 29 and Figure 30, respectively.

The results in the case of LC2 concrete are characterized by a certain dispersion, which resulted from a certain heterogeneity of the LC2 mix, which in turn resulted from the conditions of preparation of the aggregate before making the concrete mixture (outside the ITB laboratory). Further research is planned in this regard.

## 4. Discussion

The performed tests allowed us to determine the above-presented strength and long-term parameters of concretes with lightweight aggregate derived from ashes. The entire research process includes readings taken over a period of approximately three years from the time the mixtures were prepared, although the current paper describes the results obtained over a period of just over a year and a half. The empirical mutual relations between the secant modulus of elasticity of concrete and the compressive strength as well as the development of these parameters over time were also determined.

The latest research carried out at the ITB Laboratory of Building Structures, Geotechnics, and Concrete confirmed the expediency of using lightweight concrete with a special ceramic, sintered aggregate derived from ashes for prestressed structures. Two types of lightweight concrete were tested, with the W/C ratio for the LC1 mix being equal to 0.4 and that for the LC2 mix being equal to 0.5, obtained by slightly modifying the mixture compositions. These tests allowed us to determine the strength parameters of this concrete after 7, 14, 28, 60, 120 and 300 days. The compressive strength of the discussed lightweight concrete after 28 days was 57–58 MPa, according to the tests. As expected, the LC1 concrete mix obtained slightly higher strength and lower shrinkage than the LC2 concrete mix. The obtained concrete secant modulus of elasticity, amounting to approx. 25 GPa after 28 days, is slightly lower than in the case of an analogous plain concrete, but the influence of this parameter is compensated by the lower volume weight of the lightweight concrete in a finished structure. The results obtained in terms of short-term features do not differ significantly from the results presented in [8,9,13], which was confirmed in previous works by the authors [52,53].

Significant differences, however, concern the long-term characteristics. In terms of shrinkage tests, the values obtained for the concrete age of about 116 days for both the LC1 and LC2 concretes are almost twice as high as in the cited works [8,9,13], and they are higher than the values appropriate for plain concrete which, on the other hand, complies with the standards [67,72]. In terms of concrete creep strain, it was found that the average final creep coefficient for LC1 concrete samples was about 2.1 for the loading time of *t* = 419 days, and for the LC2 concrete samples it was about 1.9 for the loading time of *t* = 413 days. It can be remarked that for the concrete age of about 116 days, the values of the creep coefficient for both LC1 and LC2 concretes were approximately three times higher than in the cited works [8,9,13], but they do not differ from the values specific to plain concrete, which complies with the standard [67]. These differences result from several reasons, including different research methodologies. In this work, shrinkage and creep tests were carried out on samples by using procedures specified in the relevant standards and instructions, and creep tests were performed using dedicated equipment. On the other hand, the shrinkage and creep tests described in [8,9,13] were performed using test beams of dimensions 250 × 150 × 1500 mm. Compressive stresses were introduced in two beams of each mix (C-1 and C-2) by means of prestressing to initiate creep deformation in the concrete made of both mixes. Moreover, the stresses of the samples in the creep test were assumed in this work to be at a level of about 1/3 of the failure stress, while in the works [9,13], they were assumed to be at a level about 1.5 times lower (the initial stresses from the prestressing force ranged from 9 to 11 MPa); additionally, these stresses were difficult to estimate and changed over time. Nevertheless, the publications [8,9,13] are of great importance for the development of the knowledge about the short-term and long-term properties of new types of LWACs and their applications in prestressed concrete structures, especially floor slabs.

The course of creep deformations is correctly modeled by code methods [67,74,75,76], albeit with appropriate correction factors that should be determined in order to minimize the sum of the squares of differences between the model estimation and the experimental results [76]. However, when comparing the results of the analyses of total deformations and creep deformations obtained based on the standard model according to EC2:2004 [67] with the results obtained based on models according to pre-standards MC 2010 [74] and MC 2020 [75] (cf. graphs in Figure 23, Figure 24, Figure 25 and Figure 26 with graphs in Figure 27, Figure 28, Figure 29 and Figure 30), it can be concluded that the modifications introduced into the EC2:2004 model do not improve the approximation of the test results in the case of the type of concrete under consideration, which is also visible in relation to the comparison of the test results and shrinkage deformation analyses.

In general, it is necessary to remark that the results presented in this article were obtained under constant laboratory conditions. Built-in concrete exposed to various external factors may change its strength and mechanical properties depending on how it was made. When using these results in practice, the method of concrete production and the actual conditions under which the concrete was laid should be taken into account because the behavior of the lightweight concrete in question is influenced by technological factors.

## 5. Conclusions

Summing up, the current research results give a broader view of the possibilities of using lightweight concrete with sintered aggregate in modern construction and the development of their strength parameters over time. This paper attempts to determine the main advantages and disadvantages of LWAC with the LSA called Certyd based on the conducted tests. The advantages include the possibility of using this concrete for the construction of prestressed floor slabs, because despite the lower modulus of elasticity than in the case of plain concrete, the lower density of lightweight concrete ensures smaller deflections in floor slabs. On the other hand, the disadvantages of the tested concrete include its brittleness, which limits its applications in the case of structures in which the tensile strength of concrete is important, e.g., in the case of shear or punching. The aim of further tests will be to introduce such additives to the tested concrete that could eliminate these disadvantages. However, the condition for using the discussed concrete for prestressed structures is the method of conducting long-term tests in accordance with the relevant standards and using appropriate devices.

The aggregate used in our research is highly ecological. Reusing waste materials is now an environmental priority. As a result, the reuse of ashes for the production of concrete aggregate may in the future reduce the mining of raw materials and the use of natural aggregates in construction. The preservation of natural deposits also contributes to the protection of the environment.

## Figures and Tables

**Table 1:** Characteristics of lightweight aggregates used for concrete production.

Aggregate Type (Place of Production)	Grain Density	Bulk Density	Average Concrete Strength	Maximum Concrete Strength
			*f_cm,cube_*	*f_cm,cube_*
	[kg/m^3^]	[kg/m^3^]	[MPa]	[MPa]
Certyd (Białystok)	1300–1420	550–900	20–40	90
Pollytag (Gdańsk)	1300–1450	650–900	20–40	90
Keramzyt (Mszczonów)	950–1100	400–900	15–25	40

**Table 2:** Basic properties of aggregates.

Aggregate Type (Place of Production)	Water Absorption After 24 h	Crushing Resistance	Fractions
	[%]	[MPa]	[mm]
Certyd (Białystok)	15–20	4.0–7.0	0/2; 1/4; 4/10
Pollytag (Gdańsk)	20–25	6.0–10.0	0/4; 0.5/4; 2/5; 4/8; 6/12
Keramzyt (Mszczonów)	20–30	2.0–6.0	0/4; 3/10; 8/16; 16/31.5

**Table 3:** Concrete mixtures similar to those given in article [13].

Component	LC1 Mix	LC2 Mix
	Volume [kg/m^3^]	
Cement CEM I 42.5 N	409	419
Lightweight sintered aggregate Certyd 4/10	775	802
Sand	682	703
Water	164	209
Admixture BV 18 (plasticizer)	3.7	3.8
Admixture SKY 686 (superplasticizer)	3.7	3.8

**Table 4:** Features of fresh mixes.

Tested Feature	LC1 mix	LC2 mix
Average consistency by cone fall method (slump test) acc. to EN 12350-2 [55]	145 mm	105 mm
Consistency class acc. to EN 206 [56]	S3 class	S3 class
Average air content acc. to EN 12350-7 [57]	4.35%	4.20%
Fresh concrete mix density acc. to EN 12390-6 [58]	1960 kg/m^3^	1980 kg/m^3^

**Table 5:** Summary of LC1 test results with measurement uncertainty *U* for *t* = 419 days.

Sample No.	*ε_tot_* ± *U*	*ε_e_* ± *U*	*ε_c_* ± *U*
	[mm/m]	[mm/m]	[mm/m]
LC1-1	2.55 ± 0.07	0.72 ± 0.04	1.54 ± 0.07
LC1-2	2.66 ± 0.07	0.76 ± 0.04	1.61 ± 0.07
LC1-3	2.56 ± 0.07	0.74 ± 0.04	1.53 ± 0.07
Average value	2.59	0.74	1.56
Standard deviation	0.061	0.020	0.044

**Table 6:** Summary of LC2 test results with measurement uncertainty *U* for *t* = 413 days.

Sample No.	*ε_tot_* ± *U*	*ε_e_* ± *U*	*ε_c_* ± *U*
	[mm/m]	[mm/m]	[mm/m]
LC2-1	2.92 ± 0.07	0.82 ± 0.04	1.65 ± 0.07
LC2-2	3.16 ± 0.08	0.87 ± 0.04	1.84 ± 0.07
LC2-3	2.64 ± 0.07	0.81 ± 0.04	1.38 ± 0.07
Average value	2.91	0.83	1.62
Standard deviation	0.260	0.032	0.231

## Data Availability

The lightweight concrete with sintered aggregate described in this article is currently used in prestressed concrete bridge structures. Information on these structures is subject to NATO restrictions due to their dual use (civil and military), and detailed source data for the construction of these bridge structures are not publicly available. For this reason, it is not possible to provide detailed source data. However, any non-sensitive data can be provided upon request.

## Abbreviations

The following abbreviations are used in this manuscript:

ITB	Instytut Techniki Budowlanej (Building Research Institute)
LSA	lightweight sintered aggregate
LWAC	lightweight aggregate concrete
IMiKB	Instytut Materiałów i Konstrukcji Budowlanych (Inst. of Building Mater. & Struct.)
W/C	water/cement ratio
LC	lightweight concrete
CEM	cement class
CEB-FIP	the merger of CEB and FIP
CEB	Euro-International Committee for Concrete
FIP	International Federation for Prestressing
*fib*	International Federation for Concrete (the merger of CEB and FIP)
MC 2010	Model Code 2010
MC 2020	Model Code 2020
EC2:2004	EN 1992-1-1:2004 Eurocode 2
EC2:2023	EN 1992-1-1:2023 Eurocode 2
